# Development of a Choline Database for Thai Food and a Population-Wide Assessment of Choline Intake in Thailand

**DOI:** 10.3390/nu18162673

**Published:** 2026-08-16

**Authors:** Thet Htoo Min, Nawarat Vongvimetee, Chayanist Wanijjakul, Nipa Rojroongwasinkul, Kunchit Judprasong, Julaluk Khemacheewakul, Noppol Leksawasdi, Pornchai Rachtanapun, Akira Sittikho, Siraphat Taesuwan

**Affiliations:** 1 Faculty of Agro-Industry, Chiang Mai University, Chiang Mai 50100, Thailand; 2 Institute of Nutrition, Mahidol University, Nakhon Pathom 73170, Thailand; 3 Center of Excellence in Agro Bio-Circular-Green Industry (Agro BCG), Faculty of Agro-Industry, Chiang Mai University, Chiang Mai 50100, Thailand; 4 Nutrition and Biochemical Medicine, Department of Medicine, Faculty of Medicine Ramathibodi Hospital, Mahidol University, Bangkok 10400, Thailand; 5 Functional Foods and Nutrition Research (FFNR) Laboratory, Food, Chemical and Biotechnology Cluster, Singapore Institute of Technology, Singapore 828608, Singapore

**Keywords:** choline, dietary reference intake, food composition database, nutrition, one-carbon nutrient

## Abstract

**Background/Objectives**: Choline is an essential nutrient, and deficiency is associated with metabolic dysfunction-associated steatotic liver disease. Research shows that Western populations have inadequate choline intake; however, the intake in Asian populations is less studied. This study developed a choline database for Thai food and evaluated intake in the Thai population. **Methods**: Following guidelines of the Food and Agriculture Organization/the International Network of Food Data Systems, 1806 Thai food items were matched to the U.S. choline database, and the missForest imputation method was used to impute missing data. Choline intake was assessed in Thai adults aged 18 years and older using a nationally representative sample of the Thai Food Consumption Survey (*n* = 3593). **Results**: Animal-based foods such as eggs, meat, and fish contained more choline than plant-based foods. Median choline intake was 134.7 mg/day (IQR: 70.6, 229.3), with 3.6% of Thai individuals meeting adequate intake levels. Men consumed more choline than women (median 153 vs. 120 mg/day; *p* < 0.001). Choline intake declined with age but did not differ by body mass index. **Conclusions**: This study created a choline database for Thai food and shows that most Thais have intake below the recommended intake levels.

## 1. Introduction

Choline is an essential nutrient necessary for human health. Choline deficiency is associated with metabolic dysfunction-associated steatotic liver disease, a common liver disease [1,2]. Choline plays a crucial role in maintaining liver health by facilitating lipid transport, preserving cell membrane structure, and promoting methyl group metabolism. Choline deficiency can lead to liver damage and fat accumulation [1], while supplementation has been shown to protect and restore liver function in animals and humans [3,4]. Mixed research has shown that higher dietary choline intake tended to be associated with a reduced risk of hypertension [5,6], and was associated with reduced risks of cardiovascular diseases [7,8] and mortality [7].

Given the well-documented consequences of choline deficiency, several countries have assessed their populations’ intake of this nutrient. The Adequate Intake (AI) levels, defined as observed levels of estimated intake in a healthy population when insufficient data exist to establish the Estimated Average Requirement, were set for choline by the US Institute of Medicine (now the National Academy of Medicine) in 1998 [9]. The US choline AI levels are 550 mg/day for men and 425 mg/day for women aged 19 years and older. In 2016, the European Food Safety Authority set the AI at 400 mg/day for adult men and women aged 18 years and older [10]. Studies in the US, the EU, the UK, and Australia indicate that most individuals did not achieve the AI for choline [11,12]. In the US, 90% of the population did not meet the AI for choline [12,13,14]. Similarly, in Australia, the average daily intake was 265 mg/day, and fewer than 10% of the population met the AI levels [15]. Reports of national-level choline intake in Asia are still limited. In the Chinese population, average choline intake was 269 mg/day in men and 240 mg/day in women [16], which is also well below China’s AI levels of 450 mg/d for men and 380 mg/d for women [17]. Our previous study, conducted with a small group of Thai participants, yielded similar findings [18]. Overall, current evidence suggests that populations worldwide do not consume sufficient choline. However, the status of choline intake in Thailand remains unknown.

To evaluate choline intake in a population, two country-specific components are needed: the recommended intake (AI) and a food choline database. The AI levels for choline have been established in many countries, including Thailand, which recommends 425 mg/day for adult women and 550 mg/day for adult men [19]. The second component is a country-specific choline database, which is used to translate food consumption data into estimated nutrient intake. Currently, two major country-specific food choline databases are known. The United States Department of Agriculture (USDA)’s choline database is widely used in nutritional research to assess dietary choline intake and identify primary food sources of choline across diverse populations [12]. The second database, the Australian AUSNUT Choline Database [15], was compiled by matching food items with the USDA choline database and other published sources in accordance with the Food and Agriculture Organization of the United Nations and the International Network of Food Data Systems (FAO/INFOODS) Guidelines [20]. There is no evidence of nationwide food choline databases other than those in the US and Australia.

Given the absence of a dedicated food choline database in Thailand, accurately evaluating population intake and identifying choline-rich foods becomes challenging, thereby complicating dietary planning. Moreover, this limitation hinders policymakers from developing effective nutritional policies and food labeling. Therefore, this study aims to develop a food choline database for Thailand and assess choline intake in a nationally representative sample of the Thai population.

## 2. Materials and Methods

### 2.1. Food Matching Protocol Development

A Standard Operating Procedure for matching items in the Thai Food Composition Database with the USDA National Nutrient Database for Standard Reference, Release 28 [21], was developed in accordance with international guidelines from the FAO/INFOODS [20]. The USDA database was chosen because it provides the most comprehensive information on food components, including choline. The matching process is shown in Supplemental Figure S1. Briefly, a food item was matched to food(s) in the USDA database based on the name and the descriptors. A precise match was established when food descriptions were either identical or very similar. When no exact match was found, the closest matches, which could involve different cooking methods or mixed parts, were noted with a lower confidence grade. Nutrient retention factors from the USDA Table of Nutrient Retention Factors, Release 6, were applied to raw foods [22]. When no data were available for a selection of foods, missing-data imputation was used as a last resort. At each step, a confidence code was assigned to each food item to reflect how closely it matched another food. These confidence codes range from A, indicating a high-quality exact match, to D, indicating a weak match. Once match(es) had been found, the choline values were adjusted according to protein content using the formula $\frac{\mathrm{protein}\;\mathrm{content}\;\mathrm{of}\;\mathrm{food}\;\mathrm{in}\;\mathrm{Thai}\;\mathrm{food}\;\mathrm{database}}{\mathrm{protein}\;\mathrm{content}\;\mathrm{of}\;\mathrm{food}\;\mathrm{in}\;\mathrm{USDA}\;\mathrm{database}}\times \;\mathrm{choline}\;\mathrm{value}$. This is because previous studies suggest that food protein content correlates with choline [14]. The Standard Operating Procedure was confirmed by two personnel, who graded 20 food items from each food group during a trial run. The Standard Operating Procedure was revised until the two parties reached an agreement or was finalized by the principal investigator.

### 2.2. Development of a Food Choline Composition Database

The Standard Operating Procedure was applied to the Thai Food Composition Database developed by the Institute of Nutrition, Mahidol University, Thailand. Foods with minimal choline content according to the literature, such as fruits, sweets, and snacks, were assigned a value of 0. Eight food groups, comprising 1806 items, were selected for food matching. These groups included meat (*n* = 301), egg (*n* = 58), fish (*n* = 224), milk (*n* = 222), cereals (*n* = 334), nuts and seeds (*n* = 90), roots (*n* = 52), and vegetables (*n* = 525), standardized to 100 g edible portions. Each food item was identified by a unique code and detailed description, including preparation methods. Using this procedure, 1806 choline-containing food items were matched successfully in the Thai Food Composition Database.

### 2.3. Population-Wide Choline Intake Assessment

The choline food database was used to analyze Thailand’s national food consumption data. The Food Consumption Data of Thailand was a cross-sectional survey commissioned by the National Bureau of Agricultural Commodity and Food Standards and conducted by the Institute of Nutrition, Mahidol University, from 2013 to 2016 [23]. Participants (*n* = 8478) were chosen through a multi-stage cluster sampling method to ensure the sample was representative of the country’s population. The current study sample included individuals aged 18 years and older who completed one 24-h dietary recall (*n* = 3593). Trained personnel administered the interview using a 4-step multiple-pass technic and recorded details of all foods and beverages (time, types/descriptions, amounts, sources, and places of consumption) that the participants consumed in the previous day (midnight to midnight). Food photo software was used to aid recall of food items and portion sizes. For mixed dishes, individual ingredients and amounts were recorded [23].

The current study was approved by the Chiang Mai University Research Ethics Committee (CMUREC No. 66/252).

### 2.4. Statistical Analysis

Descriptive statistics were used to examine choline content across food categories. Specifically, the median and interquartile range (IQR) were calculated for each food group. The Random Forest imputation method (missForest R package version 4.5.3; Vienna, Austria) was applied to address missing data. This was done by training machine learning algorithms on the available observed data. Previous research has demonstrated that this technic consistently and significantly outperformed traditional approaches, such as calculating the mean or median [24]. The missForest imputation method has also been effectively applied to national food composition databases, yielding the smallest imputation errors across all tested scenarios [24].

Dietary choline intake data were observed to be characteristically right-skewed, as most of the Thai population consumed modest amounts. A small group of high-consumers contributed extreme values. To prevent results from being distorted by these outliers and to account for the non-normal distribution, non-parametric rank-based statistics were employed.

Continuous variables were reported as medians with IQRs, representing the 25th and 75th percentiles. The Kruskal–Wallis test was performed to explore whether the distribution of choline intake was similar across different comparison groups. After a significant Kruskal–Wallis result was noted, Dunn’s test was utilized as a post-hoc analysis to identify specific pairwise differences between groups. Furthermore, compliance with nutritional standards was explored by comparing median intakes against established Thai AI levels using the Wilcoxon signed-rank test [25]. All statistical computations and data visualizations were performed using R version 4.5.3 (Vienna, Austria).

## 3. Results

### 3.1. Food Composition Data Availability and Missing Data Imputation in the Thai Food Composition Database

Available data on eight food groups in the Thai Food Composition Database were matched with the USDA database to obtain choline values. Matched items are presented in Table 1. Choline values were assigned to 1294 food items: meat (214 items), eggs (49 items), fish (224 items), milk (196 items), cereals (259 items), nuts and seeds (40 items), starchy roots/tubers (36 items), and vegetables (276 items). This represents 71.7% coverage, leaving 28.3% of missing data. A Random Forest method was used to impute missing choline values across food groups, showing alignment between actual (black dots) and imputed (red dots) data (Supplemental Figure S2). Choline content varied most in meats (min = 8.1, max = 1104.9 mg/100 g), eggs (min = 0.8, max = 732.3 mg/100 g), and fish (min = 23.9, max = 435.4 mg/100 g), whereas cereals, legumes, nuts and seeds, starchy roots and tubers, and vegetables showed narrower, uniform ranges and consistent choline contributions (cereals: min = 0.2, max = 201.9 mg/100 g; legumes, nuts, and seeds: min = 2.8, max = 165.5 mg/100 g; starchy roots and tubers: min = 0.3, max = 99.9 mg/100 g; vegetables: min = 0.0, max = 118.2 mg/100 g).

### 3.2. Comparative Analysis of Choline Content in Major Food Groups

**Figure 1:**
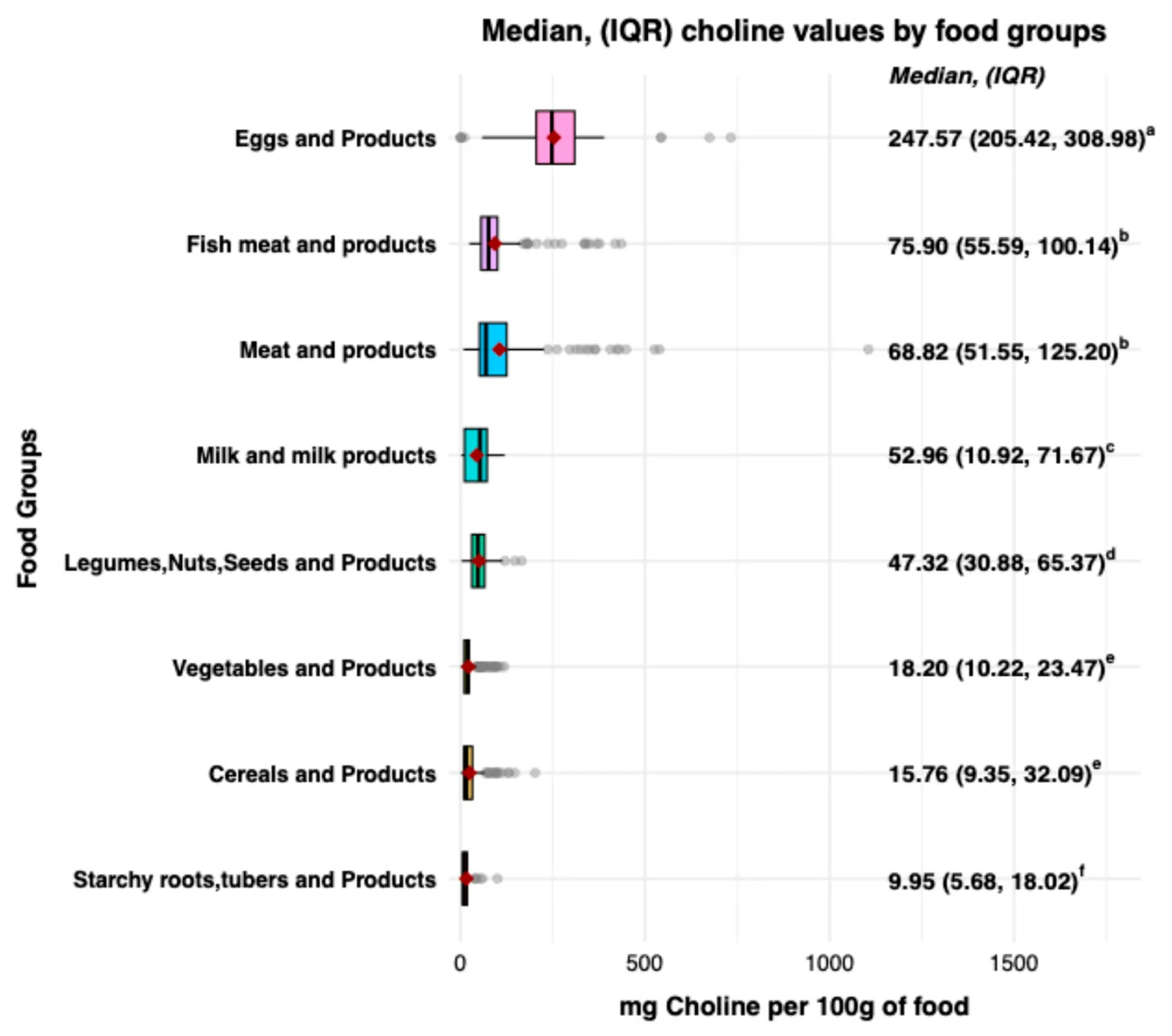
Distribution of choline content among Thai food groups. Differing letters indicate statistical significance (*p* < 0.05) based on the Kruskal–Wallis test and Dunn’s post-hoc comparison. Red diamonds indicate arithmetic means. Box plots indicate Q1 (left edge), median, and Q3 values (right edge). Left whiskers indicate Q1-1.5*interquartile range (IQR); right whiskers indicate Q3+1.5*IQR. Gray dots indicate outliers.

Comparison of choline content across food groups revealed a distinction between animal- and plant-based foods (Figure 1). Animal-derived foods had higher median choline content and IQRs than plant-based foods. Eggs and egg products had the highest median choline content [247.6 mg/100 g (IQR: 205.4, 308.9)], followed by fish [75.8 mg/100 g (IQR: 55.5, 100.1)], meat [68.8 mg/100 g (IQR: 51.5, 125.1)], and milk [52.9 mg/100 g (IQR: 10.9, 71.6)]. Legumes, nuts and seeds, vegetables, cereals, and starchy roots contained 47.3 mg/100 g (IQR: 30.8, 65.3), 18.1 mg/100 g (IQR: 10.2, 23.4), 15.7 mg/100 g (IQR: 9.3, 32.1), and 9.9 mg/100 g (IQR: 5.6, 18.0), respectively. No statistically significant difference was observed between fish and meat, or between vegetables and cereals.

### 3.3. Choline Intake in the Thai Population Stratified by Sex, Age, and Body Mass Index

**Figure 2:**
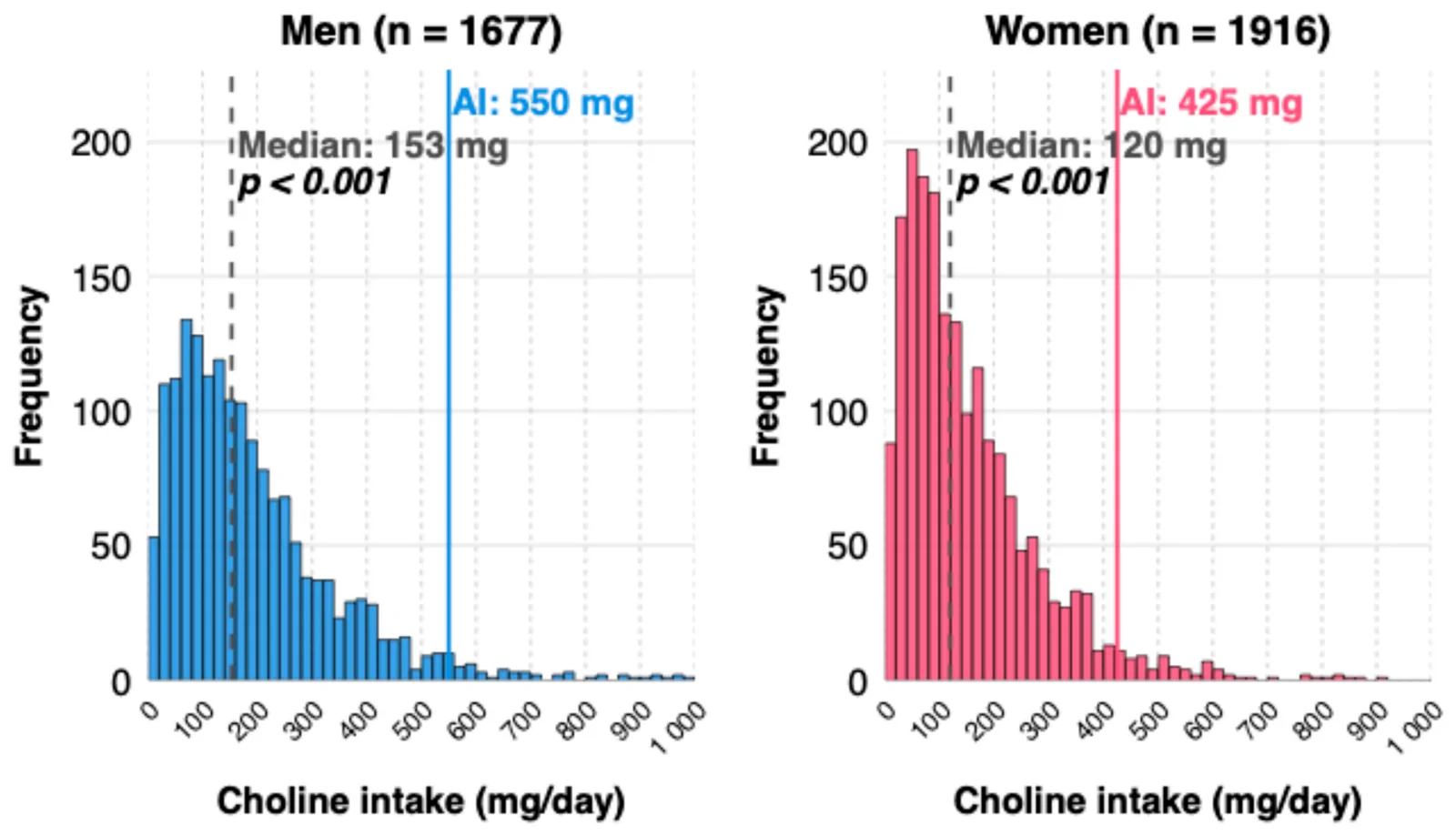
Choline intake distribution in a representative sample of Thai men and women aged 18 years and older (*n* = 3593). The histograms were derived from a single 24-h recall. The distribution may be wider than the usual intake distribution.

The developed food choline database was applied to a representative sample of 3593 Thai adults (1677 men and 1916 women) aged 18 years and older from the Food Consumption Data of Thailand [23] to determine dietary choline intake in the Thai population. Our results revealed that most Thai individuals had choline intake below the AI. The population’s median intake was 134.7 mg/day (IQR: 70.6, 229.3), with only 3.6% having intake at or above the AIs (Table 2). Both sexes had median intakes below their respective AIs (Figure 2). The median choline intake was 152.8 mg/day (IQR: 81.5, 254.1) for men and 119.5 mg/day (IQR: 62.2, 208.1) for women. Women had a lower median intake than men (*p* < 0.001), but more women achieved AI levels (4% vs. 3.1%).

**Figure 3:**
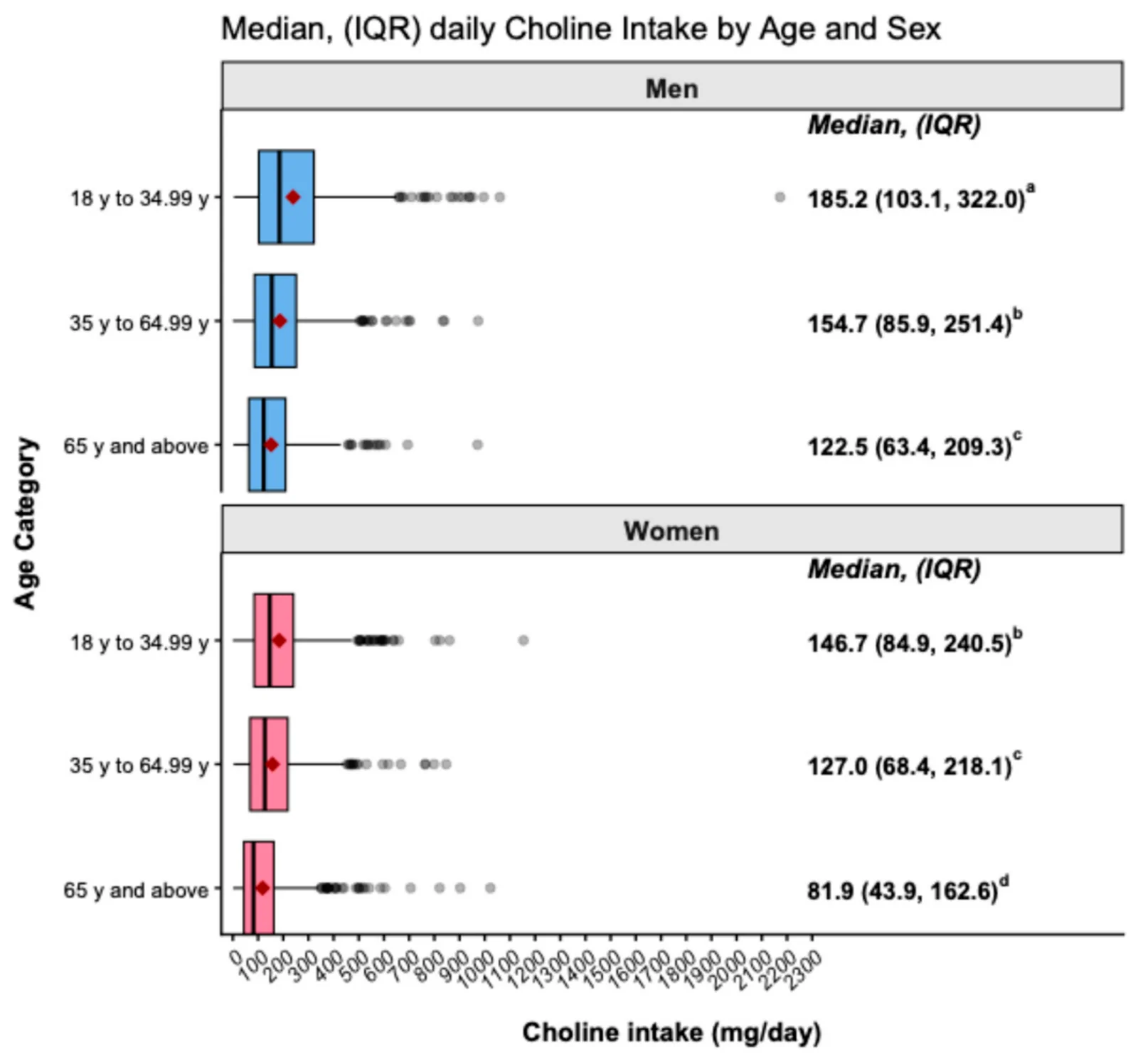
Daily dietary choline intake (mg/day) among the Thai population, stratified by age category and sex. Different letters denote a significant difference between groups.

A significant age-related decline in choline intake was observed across both sexes (Figure 3 and Table 2). Men consistently had a higher median intake than women, but all groups showed right-skewed distributions, with mostly low consumers and few high-intake outliers (Figure 3). Within each sex, choline intake declined with age. Men aged 18–34.9 years had the highest median intake (185.2 mg/day), though only 6.3% had intake at or above the AI, followed by men aged 35–64.9 years and 65 years and older, respectively. Women showed a similar pattern, with intake declining in middle age and dropping significantly among those aged 65 and older. Women aged 65 years and older had the lowest median intake, at 81.9 mg/day. The youngest-oldest gap indicated a failure to maintain nutrient density with age, and narrow IQRs were observed at the lower end, suggesting that current dietary patterns may provide insufficient choline for older people.

**Figure 4:**
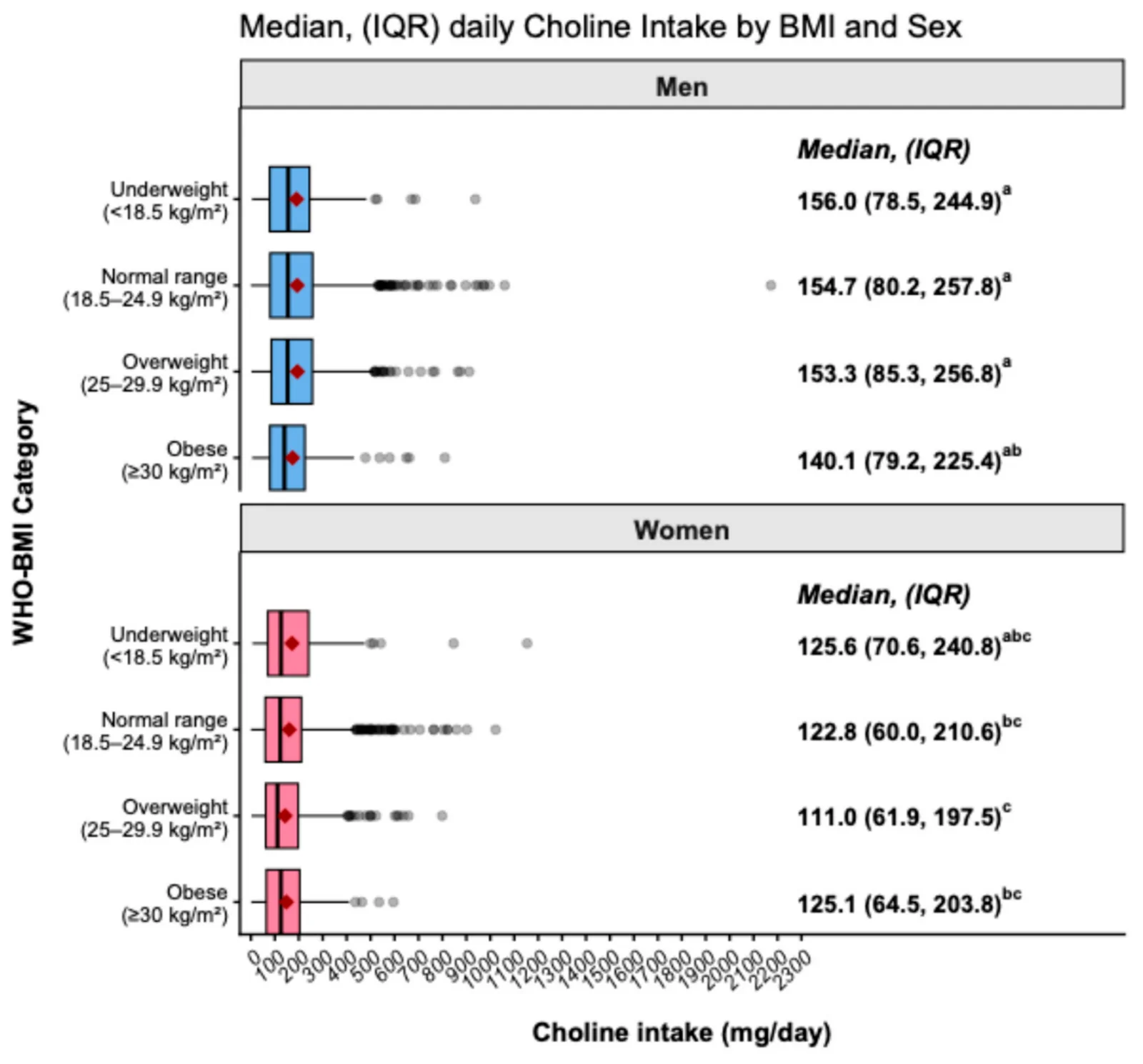
Daily dietary choline intake (mg/day) among the Thai population, stratified by WHO-BMI category and sex. Different letters denote a significant difference between groups.

When median choline intake was stratified by BMI categories and sex (Figure 4), no BMI-category differences were observed within either sex (*p* > 0.05). Underweight women had the highest proportion of intake at or above the AI (5.8%) (Table 3), and normal-weight men also showed a relatively high proportion (3.4%). Overall, our findings indicated that intake below the AIs persisted across all BMI classifications.

## 4. Discussion

To the best of our knowledge, the current study is the first to develop a choline database for foods consumed in Thailand and subsequently utilize it to assess choline intake at the population level. This database encompasses 1806 food items categorized across eight groups: meat, eggs, fish, milk, cereals, nuts and seeds, starchy roots/tubers, and vegetables. Animal-derived food generally has higher choline content than plant-derived food. Assessment of choline intake in a representative sample of the Thai adult population showed a median intake of 134.7 mg/day (IQR: 70.6, 229.3), with only 3.6% of Thai individuals consuming choline at or above the recommended AIs. Median intake in women (119.5 mg/day, IQR: 62.2, 208.1) was significantly lower than in men (152.8 mg/day, IQR: 81.5, 254.1). However, more women than men consumed choline at or above the AI levels. Higher intake was observed in younger adults (ages 18–34 years) than in their older counterparts. Across all age and BMI groups, the distribution of choline intake was right-skewed, indicating that most individuals consumed low amounts, while a small minority consumed significantly more.

Food composition data are essential for evaluating diet quality and creating food-based dietary guidelines and reference values. They identify nutrients consumed in insufficient or excessive amounts at the population level and support research into diet-disease linkages, micronutrient deficiencies, and the risk of chronic disease [26]. Their development followed global consensus and national guidelines before surveillance implementation. Food composition databases inform policy, regulation, and food labeling [27,28]. However, bias may be introduced by data gaps and outdated values, while limitations in coverage and harmonization were also found to affect intake estimate accuracy [29]. Despite these constraints, food composition databases remain important for diet-disease research, epidemiological studies, and exposure assessments [30]. In 1984, the FAO established INFOODS, a network that harmonizes food composition databases across countries and guides new database compilation [20]. Consistent with prior research, several food composition databases, including the Thai Food Composition Database, exclude choline, hindering its intake assessment. Previous dietary choline studies rely on the USDA database, which remains the most comprehensive laboratory-based source [11,21]. However, the protocol for borrowing choline values from the USDA database is often underreported.

This study introduces a flowchart-based Standard Operating Procedure for borrowing choline values from the USDA database in accordance with FAO/INFOODS guidelines. This reproducible blueprint enables other nations to systematically bridge data gaps in their national food composition repositories, thereby facilitating standardized dietary intake assessments. Nevertheless, errors attributed to differences between foods in the USDA and Thai Food Composition Databases remained and contributed to data inaccuracies. Achieving a true nutrient value for a single food is impossible in reality because foods have compositional variations, even within the same country [24]. Another issue pertinent to food composition databases is missing values. Historically, missing values were treated as zero, excluded entirely, or resolved using naive mean substitutions, which have been shown to introduce substantial errors [31]. In contrast, our study implemented a non-parametric approach by deploying Random Forest imputation via the missForest algorithm, which has been shown to be effective for handling missing nutrient values [32]. This supervised machine learning methodology accommodates both continuous and categorical variables [33], thereby preserving data variability and capturing complex, non-linear relationships among nutrient profiles.

This study provides an assessment of dietary choline intake using a representative sample of Thai adults, thereby extending surveillance to Southeast Asia. Although data on choline intake exist for Western populations, such as those in the US and Europe, as well as for East Asian populations, including China and Taiwan, this study addresses a gap in the literature concerning the Southeast Asian region. Eggs and other animal-based foods are the primary sources of choline across most age groups [15]. We found that, in general, animal-derived foods in the Thai Food Composition Database have higher choline contents and greater variability, with wider IQRs, than plant-based food groups. Using average per capita intake from the Food Consumption Data of Thailand, we estimated food group contributions and found that eggs, meat, and seafood were primary choline sources. Milk and dairy products are rarely consumed by Thai adults, resulting in minimal contribution to choline intake in the Thai diet. In contrast, dairy products significantly contribute to total choline intake in Western countries [7,15,34].

Choline intake below AIs has been reported in Western populations (the US and Europe) and in Asia, especially among women [11,12,15,16,35]. In Europe, average intakes ranged from 284 to 468 mg/day for men and 263 to 374 mg/day for women [11]. Similarly, Australian adults aged 19–64 years had average intakes of 311 mg/day for men and 248 mg/day for women [15]. Asian populations generally consume less choline than Western counterparts. In China, total choline intake ranged from 219 to 269 mg/day in men and 195 to 240 mg/day in women [16]. In the Taiwanese population, fewer than 5% of women achieved the AI [35]. Our findings align with previous Thai data, which also indicate higher intake among men than women [18]. Here, we showed that 3.6% of the Thai population consumed choline at or above the AIs. This number is likely an overestimate because it was based on single 24-h recalls, which yielded wider distributions than the true usual intake distribution.

In addition to sex, intake declined stepwise with age, suggesting that age is a primary determinant of reduced nutrient density in the Thai diet. The gap between young adulthood and older age is a critical transition period for targeted public health interventions to prevent potential age-related inadequacies. Finally, elderly women (65 years and older) reported the lowest median intake (81.9 mg/day), which is well below the AI and may require special attention given the association of choline with cognitive function preservation during aging [36].

The impact of choline deficiency on hepatic steatosis and signs of liver injury has been well documented in controlled trials involving healthy participants [37,38] and in patients receiving total parenteral nutrition [39]. A recent randomized controlled trial found that participants consuming 25% of the AI level had lower plasma choline and generally higher liver fat content than those consuming 100% AI [40]. However, circulating biomarkers of inadequate choline intake have not been established due to individual variation and non-linear relationships between diet and circulating choline levels. A cross-sectional study of 5746 middle-aged and older Norwegian adults found that total choline intake was non-linearly associated with plasma choline metabolites, and this relationship differed by sex [34]. The relationship between inadequate choline intake and hepatic fat accumulation also varies. Participants consuming 25% of the choline AI level exhibited variable liver fat deposition, with 41.6% having >10% increase in liver fat [40].

The variation in clinical signs of choline inadequacy indicates that other factors influence individual choline requirements. Genetic variations in one-carbon metabolism have been linked to the risk of organ dysfunction associated with choline deficiency (reviewed in [41]) and have influenced phosphatidylcholine production in women across different reproductive stages [42]. Currently, no study has evaluated common genetic variants in relation to circulating choline levels or choline deficiency-associated organ dysfunction in the Thai population. A small Thai study reported lower plasma folate levels in individuals with the *5,10-methylenetetrahydrofolate reductase 667CT* (*rs1801133*) genotype than in those with the *CC* genotype [43]. Yet the association between this specific polymorphism and choline status remains unclear. Overall, our study raised several important questions for Thailand: how does choline intake relate to circulating levels; what is the risk of organ dysfunction associated with choline deficiency; and how much of that risk is attributable to intake, genetics, hormonal, or other factors?

A major limitation of our study is the exclusion of data for food groups with negligible choline, including fruits, sugars, seasonings, beverages, snacks, desserts, and lipids, during food matching. These groups can collectively contribute approximately 10% to choline intake [16]; therefore, the study likely underestimated choline intake in the Thai population, especially in people who frequently consumed these food groups. Future refinements using direct analytical measurements or existing databases for these minor groups are recommended to obtain more precise population estimates. The lack of analytical validation of food items included in the database also affects the validity of the findings. Additionally, missing data are an inherent issue in food composition databases and lead to underestimated nutrient intake in the Thai Food Composition Database, although we attempted to address this issue through imputation. The missForest imputation has been validated in a database with up to 40% missing values, so the predicted estimates for the vegetables and nuts & seeds groups may be less reliable than the others. Another limitation is that reliance on single 24-h recalls yielded wider distributions than true usual intake distributions. Finally, the lack of circulating choline metabolite or liver fat measurements precluded investigation of the clinical impacts of low intake.

## 5. Conclusions

In conclusion, this study created a choline database for Thai food covering eight major food groups and 1806 items. In the Thai Food Composition Database, animal-based foods have higher, more variable choline than plant-based foods. Most Thai men and women have intake below the AI levels, although inadequacy cannot be determined based on the AI. Intake varies by sex and age: men consume more than women, and younger adults more than older adults, but intake did not differ by BMI. These findings may suggest potential under-consumption in the Thai population and a need for dietary strategies, such as educational programs to encourage consumption of choline-sourced food. Integrating the choline database into national nutrient databases, such as the Thai Food Composition Database, establishes a foundation for nutrition surveillance, dietary assessment, and policy formulation. Future research should improve food composition data, expand datasets, investigate the extent and impact of choline inadequacy in clinical settings, and develop interventions to increase intake among vulnerable groups. 

## Figures and Tables

**Table 1:** Choline values assigned to food items in select food groups of the Thai Food Composition Database.

Food Group	No. of Food Items with Choline Values/Total Foods	Proportion of Food with Choline Values (%)	Missing Values (%)
Meat	214/301	71.09	28.91
Egg	49/58	84.48	15.52
Fish	224/224	100.00	0.00
Milk	196/222	88.29	11.71
Cereals	259/334	77.54	22.46
Legumes, nuts and seeds	40/90	44.44	55.56
Starchy roots/tubers	36/52	69.23	30.77
Vegetables	276/525	52.57	47.43
Total	1294/1806	71.65	28.35

**Table 2:** Choline intake in the Thai population (*n* = 3593) by sex and age groups.

	*n*	Median (mg/day)	25th, 75th Percentile	*p*-Value (Median vs. AI)	*n* (at or Above AI) *	% (at or Above AI) *
Overall	3593	134.69	70.58, 229.33	<0.0001	128	3.56
Women						
18–34.9 years	621	146.69	84.87, 240.49	<0.0001	39	6.28
35–64.9 years	725	126.97	68.41, 218.15	<0.0001	22	3.03
65+ years	570	81.90	43.89, 162.59	<0.0001	15	2.63
Total women	1916	119.51	62.18, 208.07	<0.0001	76	3.97
Men						
18–34.9 years	539	185.24	103.07, 321.97	<0.0001	34	6.31
35–64.9 years	562	154.74	85.93, 251.36	<0.0001	11	1.96
65+ years	576	122.45	63.40, 209.28	<0.0001	7	1.22
Total men	1677	152.78	81.45, 254.06	<0.0001	52	3.10

* The number and percentage of individuals consuming at or above the adequate intake (AI) levels were derived from a single 24-h recall and may not reflect usual intake distribution.

**Table 3:** Choline intake in the Thai population (*n* = 3593) by sex and BMI (kg/m^2^).

	*n*	Median (mg/day)	25th, 75th Percentile	*p*-Values (Median vs. AI)	*n* (at or Above AI) *	% (at or Above AI) *
Women						
Underweight (<18.5 kg/m^2^)	173	125.57	70.64, 240.84	<0.0001	10	5.78
Normal (18.5 to <25 kg/m^2^)	932	122.82	59.96, 210.64	<0.0001	49	5.26
Overweight (25 to <30 kg/m^2^)	540	111.03	61.94, 197.46	<0.0001	13	2.41
Obese (≥30 kg/m^2^)	255	125.06	64.49, 203.79	<0.0001	4	1.57
Men						
Underweight (<18.5 kg/m^2^)	167	156.00	78.50, 244.92	<0.0001	3	1.80
Normal (18.5 to <25 kg/m^2^)	910	154.74	80.20, 257.81	<0.0001	31	3.41
Overweight (25 to <30 kg/m^2^)	437	153.31	85.31, 256.77	<0.0001	13	2.97
Obese (≥30 kg/m^2^)	148	140.10	79.24, 225.38	<0.0001	4	2.70

* The number and percentage of individuals consuming at or above the adequate intake (AI) levels were derived from a single 24-h recall and may not reflect usual intake distribution.

## Data Availability

The datasets presented in this article are not readily available because the Thai Food Composition Database is managed by the Institute of Nutrition at Mahidol University. The Food Consumption Data of Thailand is owned by the National Bureau of Agricultural Commodity and Food Standards. Requests to access the datasets should be directed to the respective agencies.

## Supplementary Materials

The following supporting information can be downloaded at: https://www.mdpi.com/article/10.3390/nu18162673/s1, Supplemental Figure S1. A flow diagram of choline imputation and missing data imputation; Supplemental Figure S2. Scatter plots of actual versus imputed choline values using Random Forest (missForest) in R.

## Abbreviations

The following abbreviations are used in this manuscript:

AI	Adequate intake
FAO/INFOODS	Food and Agriculture Organization of the United Nations and the International Network of Food Data Systems
IQR	Interquartile range
USDA	United States Department of Agriculture

## References

[1] Sherriff J.L., O’Sullivan T.A., Properzi C., Oddo J.-L., Adams L.A. (2016). Choline, Its Potential Role in Nonalcoholic Fatty Liver Disease, and the Case for Human and Bacterial Genes. Adv. Nutr..

[2] Guerrerio A.L., Colvin R.M., Schwartz A.K., Molleston J.P., Murray K.F., Diehl A., Mohan P., Schwimmer J.B., Lavine J.E., Torbenson M.S. (2012). Choline Intake in a Large Cohort of Patients with Nonalcoholic Fatty Liver Disease. Am. J. Clin. Nutr..

[3] Al Rajabi A., Castro G.S.F., da Silva R.P., Nelson R.C., Thiesen A., Vannucchi H., Vine D.F., Proctor S.D., Field C.J., Curtis J.M. (2014). Choline Supplementation Protects against Liver Damage by Normalizing Cholesterol Metabolism in Pemt/Ldlr Knockout Mice Fed a High-Fat Diet. J. Nutr..

[4] Sedhom S.S., El Wakeel L.M., Barakat E.M.F., Shousha H.I., Shamkh M.A., Salama S.H., Zaky D.Z., El Kholy A.A. (2025). The Impact of Choline Supplementation on Oxidative Stress and Clinical Outcomes among Patients with Non-Alcoholic Fatty Liver Disease: A Randomized Controlled Study. Ther. Adv. Chronic Dis..

[5] Taesuwan S., Thammapichai P., Ganz A.B., Jirarattanarangsri W., Khemacheewakul J., Leksawasdi N. (2021). Associations of Choline Intake with Hypertension and Blood Pressure among Older Adults in Cross-Sectional 2011-2014 National Health and Nutrition Examination Survey (NHANES) Differ by BMI and Comorbidity Status. Br. J. Nutr..

[6] Taesuwan S., Vermeylen F., Caudill M.A., Cassano P.A. (2019). Relation of Choline Intake with Blood Pressure in the National Health and Nutrition Examination Survey 2007–2010. Am. J. Clin. Nutr..

[7] Jieru P., Zhang S., Cai L., Long W., Wang Y., Zhang L., Dong Y., Zhang W., Liao J., Yang C. (2024). Dietary Choline Intake and Health Outcomes in U.S. Adults: Exploring the Impact on Cardiovascular Disease, Cancer Prevalence, and All-Cause Mortality. J. Health Popul. Nutr..

[8] Zhou R., Yang M., Yue C., Shi Y., Tan Y., Zha L., Zhang J., Chen S. (2023). Association between Dietary Choline Intake and Cardiovascular Diseases: National Health and Nutrition Examination Survey 2011–2016. Nutrients.

[9] Institute of Medicine (1998). Dietary Reference Intakes for Thiamin, Riboflavin, Niacin, Vitamin B6, Folate, Vitamin B12, Pantothenic Acid, Biotin, and Choline.

[10] Louis Bresson J., Burlingame B., Dean T., Fairweather-Tait S., Heinonen M., Ildico Hirsch-Ernst K., Mangelsdorf I., McArdle H., Naska A., Neuhäuser-Berthold M. (2016). Dietary Reference Values for Choline. EFSA J..

[11] Vennemann F.B.C., Ioannidou S., Valsta L.M., Dumas C., Ocké M.C., Mensink G.B.M., Lindtner O., Virtanen S.M., Tlustos C., D’Addezio L. (2015). Dietary Intake and Food Sources of Choline in European Populations. Br. J. Nutr..

[12] Zuk E., Nikrandt G., Chmurzynska A. (2024). Dietary Choline Intake in European and Non-European Populations: Current Status and Future Trends-a Narrative Review. Nutr. J..

[13] Papanikolaou Y., Fulgoni V.L. (2021). Patterns of Egg Consumption Can Help Contribute to Nutrient Recommendations and Are Associated with Diet Quality and Shortfall Nutrient Intakes. Nutrients.

[14] Wallace T., Fulgoni V. (2017). Usual Choline Intakes Are Associated with Egg and Protein Food Consumption in the United States. Nutrients.

[15] Probst Y., Guan V., Neale E., Probst Y., Guan V., Neale E. (2019). Development of a Choline Database to Estimate Australian Population Intakes. Nutrients.

[16] Chen P., Wu S., Tan P., Sui Y., Lu J., Peng T., Wang W., Lu W., Zhu H., Li K. (2025). Trends in Dietary Choline and Betaine Intake among Chinese Adults: The China Health and Nutrition Survey 1991-2011. Br. J. Nutr..

[17] Chinese Nutrition Society. https://en.cnsoc.org/DRIs/122510202.html.

[18] Jindasereekul P., Jirarattanarangsri W., Khemacheewakul J., Leksawasdi N., Thiennimitr P., Taesuwan S. (2023). Usual Intake of One-Carbon Metabolism Nutrients in a Young Adult Population Aged 19–30 Years: A Cross-Sectional Study. J. Nutr. Sci..

[19] Dietary Reference Intake for Thais Revision Task Force and Committee (2020). Dietary Reference Intake for Thais 2020.

[20] Charrondiere U.R., Rittenschober D., Nowak V., Stadlmayr B., Wijesinha-Bettoni R., Haytowitz D. (2016). Improving Food Composition Data Quality: Three New FAO/INFOODS Guidelines on Conversions, Data Evaluation and Food Matching. Food Chem..

[21] Agricultural Research Service USDA National Nutrient Database for Standard Reference, Release 28 (Slightly Revised). https://www.ars.usda.gov/northeast-area/beltsville-md-bhnrc/beltsville-human-nutrition-research-center/methods-and-application-of-food-composition-laboratory/mafcl-site-pages/sr11-sr28/.

[22] Nutrient Data Lab (2007). USDA Table of Nutrient Retention Factors, Release 6.

[23] National Bureau of Agricultural Commodity and Food Standards (2016). Food Consumption Data of Thailand.

[24] Ispirova G., Eftimov T., Seljak B.K. (2020). Evaluating Missing Value Imputation Methods for Food Composition Databases. Food Chem. Toxicol..

[25] Mangiafico S.S. (2015). An R Companion for the Handbook of Biological Statistics.

[26] Elmadfa I., Meyer A.L. (2010). Importance of Food Composition Data to Nutrition and Public Health. Eur. J. Clin. Nutr..

[27] Durazzo A., Lucarini M. (2025). Food Composition and Dedicated Databases: Key Tools for Human Health and Public Nutrition (2nd Edition). Nutrients.

[28] McKillop K., Harnly J., Pehrsson P., Fukagawa N., Finley J. (2021). FoodData Central, USDA’s Updated Approach to Food Composition Data Systems. Curr. Dev. Nutr..

[29] Ferraz de Arruda H., Aleta A., Moreno Y. (2023). Food Composition Databases in the Era of Big Data: Vegetable Oils as a Case Study. Front. Nutr..

[30] Delgado A., Issaoui M., Vieira M.C., Saraiva de Carvalho I., Fardet A. (2021). Food Composition Databases: Does It Matter to Human Health?. Nutrients.

[31] Balakrishna Y., Manda S., Mwambi H., van Graan A. (2022). Statistical Methods for the Analysis of Food Composition Databases: A Review. Nutrients.

[32] Ismail A.R., Abidin N.Z., Maen M.K. (2022). Systematic Review on Missing Data Imputation Techniques with Machine Learning Algorithms for Healthcare. J. Robot. Control (JRC).

[33] Stekhoven D.J., Bühlmann P. (2012). MissForest—Non-Parametric Missing Value Imputation for Mixed-Type Data. Bioinformatics.

[34] Van Parys A., Brække M.S., Karlsson T., Vinknes K.J., Tell G.S., Haugsgjerd T.R., Ueland P.M., Øyen J., Dierkes J., Nygård O. (2022). Assessment of Dietary Choline Intake, Contributing Food Items, and Associations with One-Carbon and Lipid Metabolites in Middle-Aged and Elderly Adults: The Hordaland Health Study. J. Nutr..

[35] Chu D.-M., Wahlqvist M.L., Chang H.-Y., Yeh N.-H., Lee M.-S. (2012). Choline and Betaine Food Sources and Intakes in Taiwanese. Asia Pac. J. Clin. Nutr..

[36] Chen H., Li T., Wu M., Flores-Torres M.H., Zheng J., Guo Z., Yu J., Luo B., Liu D., Lu Y. (2026). Dietary Choline Intake and Incident Dementia in Middle-Aged and Older Adults: A Prospective Cohort Study and Dose-Response Meta-Analysis. Nutr. J..

[37] Zeisel S.H., Da Costa K.A., Franklin P.D., Alexander E.A., Lamont J.T., Sheard N.F., Beiser A. (1991). Choline, an Essential Nutrient for Humans. FASEB J..

[38] Fischer L.M., DaCosta K.A., Kwock L., Stewart P.W., Lu T.-S., Stabler S.P., Allen R.H., Zeisel S.H. (2007). Sex and Menopausal Status Influence Human Dietary Requirements for the Nutrient Choline. Am. J. Clin. Nutr..

[39] Buchman A.L., Ament M.E., Sohel M., Dubin M., Jenden D.J., Roch M., Pownall H., Farley W., Awal M., Ahn C. (2001). Choline Deficiency Causes Reversible Hepatic Abnormalities in Patients Receiving Parenteral Nutrition: Proof of a Human Choline Requirement: A Placebo-Controlled Trial. JPEN J. Parenter. Enter. Nutr..

[40] Trujillo-Gonzalez I., Horita D.A., Stegall J., Coble R., Paules E.M., Lulla A.A., Baah E., Bottiglieri T., Sha W., Kohlmeier M. (2026). Choline and Betaine Concentrations in Plasma Discriminate Levels of Dietary Choline Intake in Healthy Adults: Analysis of a Double-Blind Randomized Crossover Controlled Feeding Study. Am. J. Clin. Nutr..

[41] Ganz A.B., Klatt K.C., Caudill M.A. (2017). Common Genetic Variants Alter Metabolism and Influence Dietary Choline Requirements. Nutrients.

[42] Ganz A.B., Shields K., Fomin V.G., Lopez Y.S., Mohan S., Lovesky J., Chuang J.C., Ganti A., Carrier B., Yan J. (2016). Genetic Impairments in Folate Enzymes Increase Dependence on Dietary Choline for Phosphatidylcholine Production at the Expense of Betaine Synthesis. FASEB J..

[43] Chitta P., Pratumvinit B., Kaewboonruang W., Dawangpa A., Khamrangsee S., Assantachai P., Sutiwisesak R., Wongloet W., Tencomnao T., Sae-Lee C. (2025). Vitamin B Levels in Older Adults with Pre-Frailty and Frailty: The Impact of MTHFR and TCN2 Polymorphisms and Their Association with Global DNA Methylation and Physical Performance. Nutr. Metab..

