# Supplementary material for: Development of a Choline Database for Thai Food and a Population-Wide Assessment of Choline Intake in Thailand
Source: Nutrients. 2026 Aug 16;18(16):2673. doi: 10.3390/nu18162673 (PMC13516779; doi:10.3390/nu18162673)

SUPPLEMENTAL FIGURES

Supplemental Figure S1. A flow diagram of choline imputation and missing data imputation

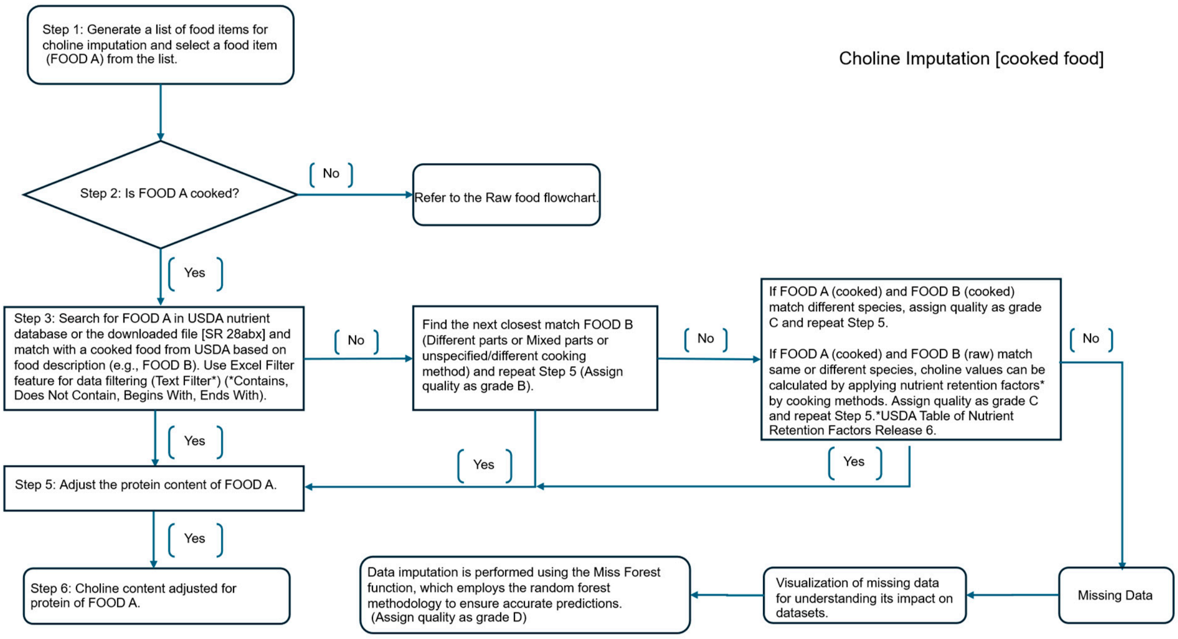

Supplemental Figure S2. Scatter plots of actual versus imputed choline values using Random Forest (missForest) in R.

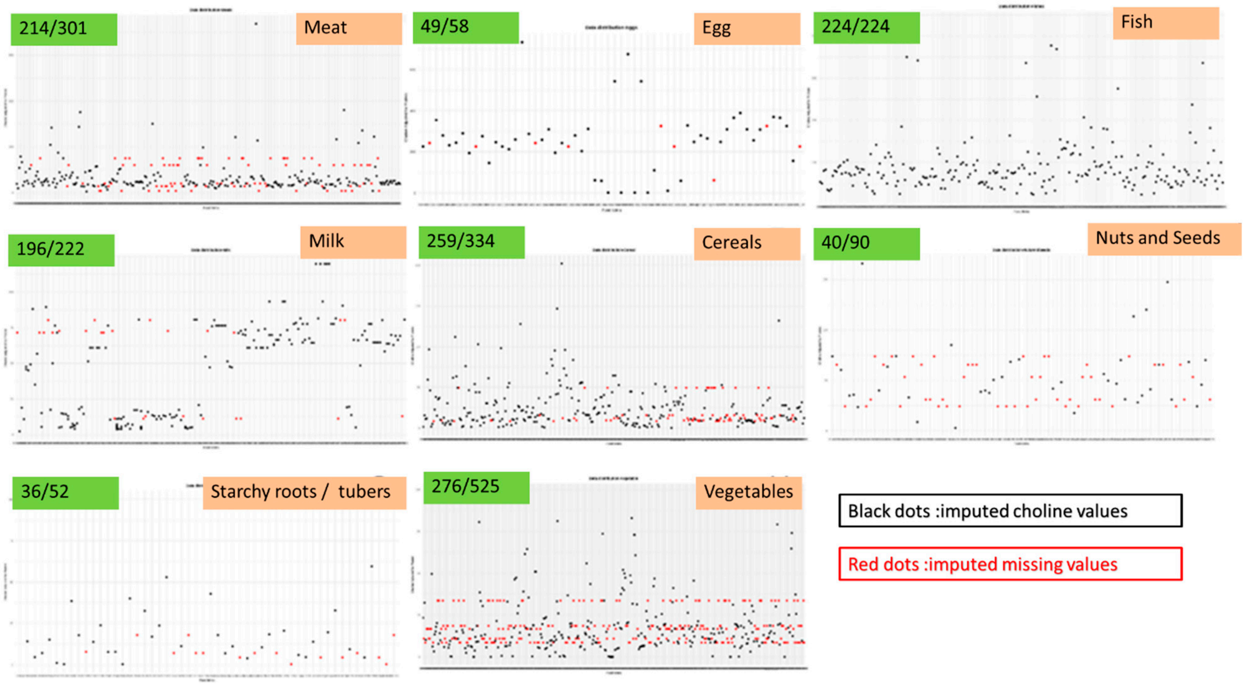

Supplement: Supplementary file 1 [file nutrients-18-02673-s001.zip › nutrients-4448046-supplementary.pdf]
